# Applications and Prospects of CRISPR/Cas9-Mediated Base Editing in Plant Breeding

**DOI:** 10.3390/cimb45020059

**Published:** 2023-01-19

**Authors:** Yizhen Li, Jing Liang, Bufang Deng, Yingli Jiang, Jingyan Zhu, Like Chen, Min Li, Juan Li

**Affiliations:** 1 College of Agronomy, Anhui Agricultural University, Heifei 230036, China; 2 Key Laboratory of Rice Genetic Breeding of Anhui Province, Rice Research Institute, Anhui Academy of Agricultural Sciences, Hefei 230031, China

**Keywords:** base editing, CRISPR/Cas9, plant, genome editing, crop improvement

## Abstract

The clustered regularly interspaced short palindromic repeats (CRISPR)/associated protein 9 system (Cas9) has been used at length to optimize multiple aspects of germplasm resources. However, large-scale genomic research has indicated that novel variations in crop plants are attributed to single-nucleotide polymorphisms (SNPs). Therefore, substituting single bases into a plant genome may produce desirable traits. Gene editing by CRISPR/Cas9 techniques frequently results in insertions–deletions (indels). Base editing allows precise single-nucleotide changes in the genome in the absence of double-strand breaks (DSBs) and donor repair templates (DRTs). Therefore, BEs have provided a new way of thinking about genome editing, and base editing techniques are currently being utilized to edit the genomes of many different organisms. As traditional breeding techniques and modern molecular breeding technologies complement each other, various genome editing technologies have emerged. How to realize the greater potential of BE applications is the question we need to consider. Here, we explain various base editings such as CBEs, ABEs, and CGBEs. In addition, the latest applications of base editing technologies in agriculture are summarized, including crop yield, quality, disease, and herbicide resistance. Finally, the challenges and future prospects of base editing technologies are presented. The aim is to provide a comprehensive overview of the application of BE in crop breeding to further improve BE and make the most of its value.

## 1. Introduction

The clustered regularly interspaced, short palindromic repeats (CRISPR)/CRISPR-associated 9 (Cas9) is a third-generation gene editing technology following ZFNs and TALENs. It has the advantages of being highly efficient, simple, inexpensive, and easily usable [1,2,3].

In the CRISPR/Cas9 system, a Cas9-single guide RNA (sgRNA) complex binds to a specific nucleotide sequence with the guidance of the sgRNA and cleaves the target DNA strand, causing a double-strand break (DSB) [4,5,6,7]. These DSBs can be corrected by nonhomologous end-joining (NHEJ) or the homology-directed repair (HDR) mechanism [8,9]. NHEJ is a method of repair in which the ends of DSBs are directly linked by DNA ligase and do not depend on homologous DNA sequences; therefore, NHEJ repair is rapid but not exact. The homologous repair process is complex and precise but requires a homologous DNA sequence template and can occur only in the G2/S phase of a cell [10,11,12,13].

Due to the genetic basis underlying the diversity of many important crop species and single-nucleotide variations [14,15], it is necessary to develop a technique that allows precise and effective single-base substitutions. Base editing technology is a novel target gene modification technique developed based on the CRISPR/Cas system, by its utilization of a tethered deaminase domain or nickase Cas9 for base conversion from A > G or C > T or C > G without the donor DNA and a DSB introduction in the genome. Recent studies have utilized Base-editors to create single and multiple nucleotide modifications in cells. Here we review different base editing technologies and their applications in crop improvement.

## 2. Base Editing

**Figure 1:**
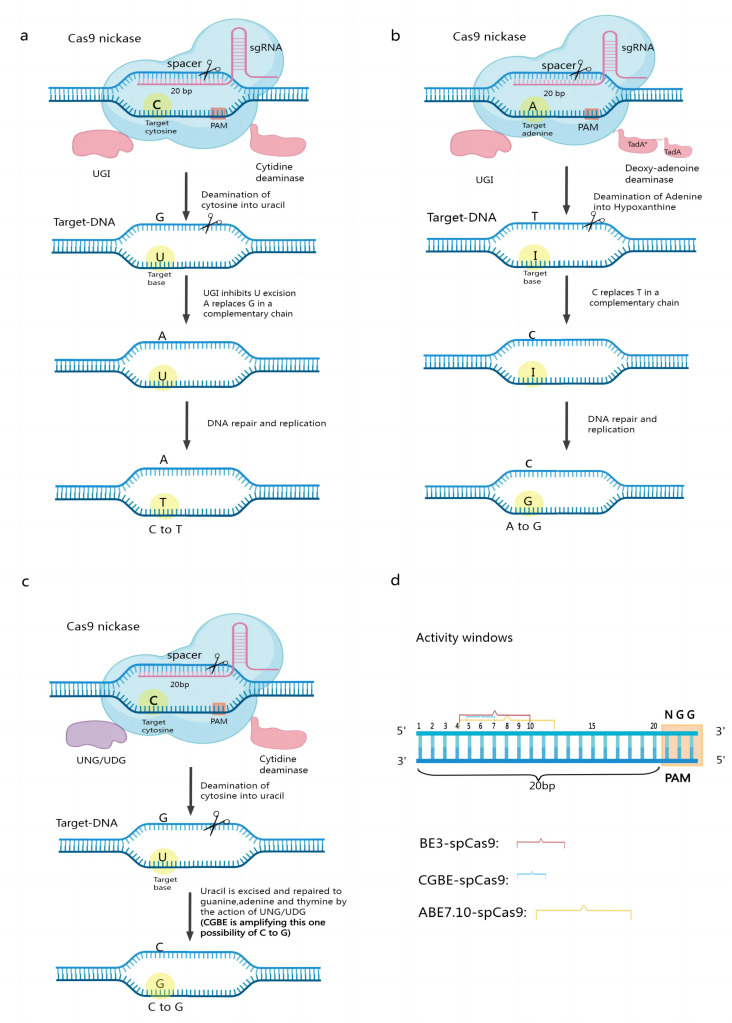
Mechanism of base editing work. (**a**) Structure and working mechanism of the cytosine base editor; (**b**) Structure and working mechanism of the adenine base editor; (**c**) Structure and editing mechanism of CGBE; (**d**) Window of activity for some typical CBEs, ABEs, and CGBEs.

Base editors (BEs) enable single-nucleotide targeted mutations without severing the nucleic acid backbone and enable direct chemical modification of target nucleobases. The original base editor used a single-stranded DNA-specific cytidine deaminase combined with an inactivated Cas9 (dCas9) to convert a cytosine (C)-guanine (G) base pair to thymine (T)-adenine (A) in the target region, called the cytosine base editor (CBE). Later researchers developed the Adenine Base Editor and the Guanine Base Editor based on the CBE. Currently available base editing systems include cytosine (C) base editors (CBEs), adenine (A) base editors (ABEs), and guanine (G) base editors (CGBEs) (Figure 1) [16,17,18,19]. Each of these categories is discussed below.

## 3. CBEs

The first-generation CBE, BE1, consists of rat C deaminase (rAPOBEC1) and dCas9, whose cleavage activity is completely lost [20,21]. When the fusion protein targets genomic DNA under the guidance of sgRNA, C deaminase can bind to the ssDNA in the R-loop region formed by the Cas9 protein, sgRNA, and genomic DNA and deaminate C to uracil (U) within a certain range along the ssDNA. During DNA replication, U is read by DNA polymerase as thymine (T). The final substitution of C/G to T/A base pairs then occurs (Figure 1a). Later researchers developed a second-generation cytosine base editor, BE2, by incorporating a uracil DNA glycosylase inhibitor (UGI) from phage PBS on top of BE1. Because UGI can inhibit the action of uracil DNA glycosylase (UDG) in the organism, BE2 is three times more efficient at editing than BE1. CBEs have undergone several generations of updates; notably, BE3 has replaced dCas9 in BE2 with nCas9(D10A) [22,23,24,25]. nCas9(D10A) specifically creates a gap in the nonedited strand, which in turn stimulates the intracellular base mismatch repair pathway (MMR) [26,27], which uses the editing strand containing U as a template for repair, resulting in increased editing efficiency. These optimized CBEs can better serve precision breeding [18,24,28]. However, existing CBEs/CGBEs rely on the natural cytosine deaminase AID/APOBEC and often produce high insertional deletion by-products and off-target effects due to the activation of the base excision repair pathway by cytosine deamination. Recently, researchers have transformed the adenine deaminase TadA-8e into a non-natural cytosine deaminase using only cytosine as a substrate and constructed the first novel CGBE/CBE family of base editors—Td-CGBE/Td-CBEs—that do not rely on the AID/APOBEC deaminase family, demonstrating lower off-target effects and very low indels events. Constructing a corresponding base editor in plants will facilitate crop improvement [29].

## 4. ABEs

Three main components compose ABEs: synthetic A deaminase, nCas9 (D10A), and sgRNA [30]. The A deaminase protein binds to ssDNA and deaminates A into inosine I, which is then read and replicated as G at the DNA level. This enables the instant exchange of A–T base pairs with G–C base pairs when the fusion protein targets genomic DNA under the guidance of sgRNA [31] (Figure 1b). Using ABEs eliminates the limitation that CBEs can edit only C or G and opens up a wider range of base transformation possibilities. In contrast to CBEs, ABEs do not require the suppression of alkyl adenine DNA glycosylase (AAG) activity [32,33,34].

## 5. CGBEs

C-to-G base editors (CGBEs) were constructed by adapting existing CBE tools to generate a new tool suitable for mediating C–G base reversals [35].

Combining a Cas9 nickase (nCas9-D10A), cytidine deaminase, and Uracil-N-glycosylase (UNG) leads to the production of CGBEs. Cytidine deaminase causes the conversion of a target C to U under the guidance of RNA. UNG locates U in the DNA and eliminates it, resulting in the formation of an AP site [36]. When nCas9 creates the AP site and binds the nonedited strand, DNA repair and replication mechanisms are triggered, preferentially inserting a G at the AP site. In contrast to CBEs, which contain a UNG inhibitor, CGBEs contain UNG [37,38] (Figure 1c).

In the base editor, the single-stranded DNA in the R-loop is exposed during base editing. This single-stranded DNA binds to the 20 bp of the sgRNA, but there is a preference for the action of cytidine deaminase on this 20 bp fragment so that different base editors have specific BE activity windows. We define the 20th base from the PAM site as the first position. The Activity Window of the Classical Cytosine Base Editor BE3-SpCas9 is bases 4–10. Bases 4–12 of the classical adenine is the base editor ABE7.10-SpCas9 activity window. Bases 5–7 of the classical guanine base editor is the CGBE-SpCas9 activity window. The different base editor activity windows depend on various factors, such as Cas proteins, deaminases, and variant connectors [39,40,41] (Figure 1d).

## 6. Application of Base Editors in Plants

In the long history of breeding, several major strategies have been used, such as crossbreeding, mutation breeding, etc. Gene editing and transgenics are an important part of the new breeding era [42,43,44,45,46,47]. Crossbreeding can only introduce known good traits [48,49]. Mutation breeding is a longer breeding process in which researchers create random mutations throughout the plant genome through physical and chemical mutagenesis. Transgenic breeding techniques allow for the direct introduction of good genes specific to a crop or genes from other species to obtain crop varieties with higher yields and better nutritional quality [50,51,52,53]. However, this breeding method requires the integration of exogenous genes into the plant genome and is, therefore, subject to strict controls. BE technology is an effective complement to the above three breeding methods. BEs allow for targeted modification of the plant genome without introducing exogenous genes to obtain the target variety quickly [54,55,56,57].

In recent years, public investment in research has been used to sequence, assemble and annotate the genomes of major crops, and we have gained a wealth of functional genetic information on plants [54,55,56,57,58]. Base editing allows for precise genome editing, and its successful operation in breeding has opened up new opportunities for crop improvement. Since 2016, BEs have been used to edit the genomes of various plant species, including rice, maize, cotton, oilseed rape, tomato, strawberry, and watermelon [59,60,61,62,63,64,65,66,67,68,69]. The contributions of BEs to improved yields, increased stress resistance, improved herbicide resistance, and quality-regulated nutrient composition of various crop species are reviewed in this paper. As traditional breeding techniques and modern molecular breeding technologies complement each other, various genome editing technologies have emerged [70,71,72]. The question of how to realize the greater potential of BE applications is one that we need to consider.

## 7. Increasing Yields

Satisfying the growing demand for increased crop yields is extremely challenging due to soil degradation, climate change, and many other constraints [73,74]. In recent years, traditional breeding has been dedicated to increasing crop yields. However, traditional breeding is accompanied by long cycle times and often by linkage resistance. Many plant genetic variants contain deleterious mutations. Mutation breeding, on the other hand, requires multigenerational crosses, backcrosses, etc. The process is time-consuming and laborious. BEs allow for precisely targeted modifications to plant genomes and can rapidly alter species traits. *OsSPL* regulates the meiotic fate in rice. With this in mind, it is possible to generate high-yielding rice lines by developing various sgRNAs and different adenylation base codons that target *OsSPL14*, *OsSPL16*, *OsSPL17*, and *OsSPL18* sequences [75,76] (Table S1). A point mutation in the OsmiR156 binding site of *OsSPL14* leads to OsmiR156-mediated cleavage of the *OsSPL14* transcript, resulting in rice plants with desirable architecture and increased seed yields [77,78,79] (Table S1). It was shown that base editing improved yields without introducing a cumbersome trait. The *OsSPL14* (which governs the desired rice structure) and *OsSIR16* (which controls rice grain size, shape, and quality) genes were simultaneously edited by the use of CBE(A3A/Y130F-CBE-V01) to generate lines with improved high expression of *OsSPL14* and *OsSPL16* (Table S1). Concerning these edited lines, we can obtain increased yields [75,80,81]. Base editing can also be used to produce higher-yielding crops using other strategies. CBE can produce C-to-T base mutations at specific sites, converting non-terminating codons to stop codons and thereby silencing gene expression. *OsGS3* is a quantitative trait locus (QTL) that regulates the rice grain length and width; *OsGW2* is a QTL that controls rice grain width and weight; regulation of the expression of *OsGS3* and *OsGW2* genes using CBE-driven insertion of premature stop codons can generate high-yielding rice lines [80,82,83,84] (Table S1). It follows that, in the case of known target genes, the application of BE in plant crops is a novel strategy to improve crop traits for more efficient and sustainable agriculture.

## 8. Improving Quality

The quality of crop products is directly related to the edibility and economic value of a crop. The amylose content (AC) is a critical determinant of the edible and culinary quality (ECQ) of rice [85,86]. Specifically, Xian (*indica*) and Geng (*japonica*), both of which are commonly consumed, comprise 10% to 20% straight-chain starch [87,88].

Rice with a medium-to-low AC concentration is often softer, with higher palatability and increased glossiness. Rice with a high AC is stiffer [89,90]. The rice *Waxy (Wx)* gene is vital for the edibility and culinary quality (ECQ) of rice. *Wx* encodes grain-bound starch synthase I (*GBSSI*), an enzyme that regulates the synthesis of straight-chain starch and ultimately determines the endosperm’s straight-chain starch content [91,92,93]. The *GBSS* allele of Xian rice variety YK17 was edited via ABEs to decrease the AC content while maintaining ECQ parameters such as gel consistency (GC) and alkali diffusion value; these traits could significantly enhance the ECQ of Xian rice variety YK17 [94] (Table S1). Similar methods were used to produce mutant Geng rice lines with a low AC content, where precise BE of the Geng *Wx* gene by CBEs controlled the overall abundance and activity of *GBSSI*. Mutant Geng rice with a low AC content was obtained [92] (Table S1). In addition, three starch branching enzymes (SBE), *OsSBEI*, *OsSBEIIa*, and *OsSBEIIb*, are included in rice. *OsSBEIIb* is specifically expressed in the endosperm and can transfer short chains to branched starch crystals. Inactivation of *OsSBEIIb* leads to reduced branching of branched starch, increased straight-chain starch content, increased seed opacity, and reduced dry weight [95,96,97]. Researchers selected two targets (S3 and S5) within the rice *OsSBEIIb* gene and successfully induced point mutations via pCXUN-BE3 (Table S1); this experiment highlighted a viable and effective tool for the modification of straight-chain starch content in rice.

BEs can be applied to other crop species and also improve traits in economically important crop species. Tomato fruit and tomato-related foods provide a significant amount of lycopene to humans [98,99,100]. The primary pigments in tomato fruit are carotenoids, such as lycopene and beta-carotene. Carotenoids are essential functional components because of their strong antioxidant ability, and *SlDDB1, SlDET1*, and *SlCYC-B* play significant roles in carotenoid accumulation. Target-AID, developed in 2016, can introduce a base substitution within a target gene. Target-AID binds PmCDA1 and dCas9 from the eel. The basic principle of Target-AID is the same as BE, but the deaminase used is different; the deaminase used in Target-AID is PmCDA1, which is in the AID family [66,101,102,103]. Targeting studies on the *SlDDB1*, *SlDET1*, and *SlCYC-B* genes have been conducted using the Target-AID technique. The findings suggested that the BE strategy could increase the carotenoid content of tomato fruit and that the altered lines presented significant changes in carotenoid accumulation [66] (Table S1). In addition, strawberry is another of the major economically important crop species. Different genotypes of strawberries had varying effects on the sugar content of their fruit, thus enhancing the genotype and trait variety. Human APOBEC3A, coupled with the Cas9 protein to increase plant base editing effectiveness, was used to generate the novel plant BE known as A3A-PBE [104]. Researchers have utilized A3A-PBE to edit the conserved uORF of the strawberry *Fv*ebZIPs1.1 gene to generate seven novel uORFs in the T0 generation. The homozygous mutants with the seven novel uORF mutations presented varying degrees of increased fruit sugar contents, and there was no effect on plant growth (Table S1). This example shows that, using the Single Base Editor, it is possible to precisely adjust the sugar content of strawberries for different production requirements [67].

## 9. Crop Morphology and Nitrogen Uptake

In addition, BE can not only be used to improve the nutrient content of crop plants but can also enhance crop morphology and regulate nitrogen uptake. *NRT1.1B* encodes a nitrogen transporter [105,106] (Table S1), and research has indicated that a C→T substitution (Thr327Met) in this gene increases nitrogen use efficiency in rice. *SLR1* encodes a DELLA protein with an amino acid substitution in or near its TVHYNP sequence, reducing plant height [107,108,109] (Table S1). Similarly, CBEs have been used to target specific regions of *IAA7* and *RGA*, which encode growth hormone response proteins and gibberellin signaling proteins, resulting in targeted mutations of C bases to T bases at the target site. Plants exhibited a dwarf-type morphology to varying degrees after mutations of single bases in *IAA7* and *RGA* (Table S1). In addition, another study showed that base editing systems could be used for the genetic improvement of kale-type oilseed rape adapted to mechanized breeding [110] (Table S1). It follows that base editing can be used for crop improvement and germplasm innovation. We can use base editing to construct sgRNA libraries to screen for mutant loci that do not exist in nature and select valuable mutant loci to improve crop quality.

## 10. Disease Resistance

Breeding stable disease-resistant plants is an economical and eco-friendly way to control crop diseases to sustain agricultural production. Rice blast is a major rice disease [111,112]. Mitigating or resolving rice blight is a problem for breeders to consider. *Pi-d2* is an agriculturally important rice blast resistance gene. Previous studies have indicated that a single amino acid substitution at position 441 of the recessive allele of the *Pi-d2* gene resulted in the loss of resistance to rice blast. CRISPR/Cas9 gene editing technology is powerless against single-base editing [113]. The use of an improved BE, hAID*Δ-XTEN-Cas9n-NLS (rBE5), was effective at rescuing resistance to rice blast in Pi-d2 mutants [114,115,116] (Table S1); rBE5 is a single-base editor for the introduction of human-derived AID cytosine deaminase. BEs can also be used in the treatment of other plant diseases. The *OsSWEET14* gene is a susceptibility gene for rice leaf blight [117,118,119]. AFID systems (APOBEC-Cas9 fusion-induced deletion systems, AFIDs) are novel polynucleotide-targeted deletion systems in which wild-type SpCas9 is included with the cytosine deaminase APOBEC, uracil glycosylase (UDG), and purine-pyrimidine site-free cleavage enzymes (AP cleavage enzymes). The AFID system converts the cytidine on the non-target strand from APOBEC deaminase to uridine; UDG then excises the uracil from the uridine to create the AP site, which is removed by AP lyase; Cas9 cuts both strands to form the DSB, which leads to a “predictable” deletion from the deaminase C extension to the DSB via the NHEJ repair pathway. Researchers have used the AFID-3 system to target the effector binding element within the promoter of the *OsSWEET14* gene in rice and obtained mutant plants in which polynucleotide sequences were deleted (Table S1); these mutants were subjected to leaf blight inoculation, and the results showed that, compared with plants in which only 1~2 bp were deleted, mutant plants in which polynucleotide sequences were deleted were more resistant to the leaf blight fungus [120]. With the development of sequencing technologies and bioinformatics, we can explore gene function more easily. More and more disease-resistant genes will be discovered, and using BE technology in combination with bioinformatics, precise editing of disease-resistant genes using BEs may be a promising approach to protect plants from biotic stresses [121].

## 11. Herbicide Resistance

Weeds compete with crop plants for nutrients, sunlight, and living space, disrupt airflow, intercept light, and promote pests and diseases in the field. Moreover, parasitic weeds absorb nutrients from crop plants, thereby decreasing the yield and quality of those crops [122,123]. Deploying weed management practices is therefore important [124,125,126].

**Figure 2:**
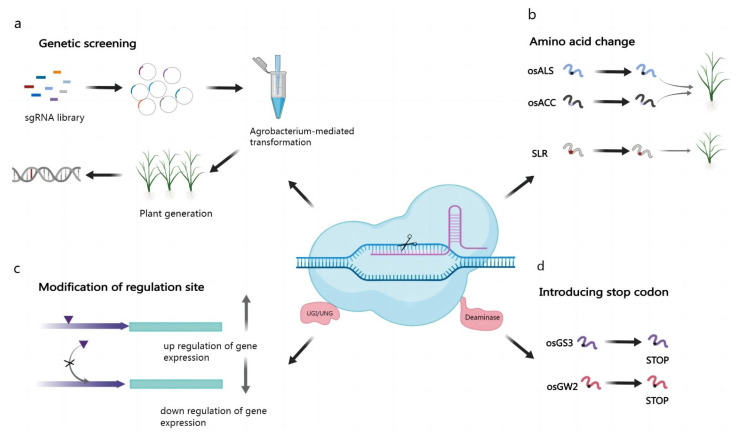
Applications of base editing technology in crops. (**a**) Construction of sgRNA libraries for gene screening; (**b**) Single base mutations causing amino acid changes and thus developing superior traits; (**c**) Editing regulatory factors to regulate gene expression; (**d**) Introduction of stop codons in advance to regulate gene expression.

BEs offer a precise and rapid way of generating new herbicide-resistant plant lines [127]. Acetyl lactate synthase *(ALS)* is a critical enzyme for synthesizing branched-chain amino acids and is an important target for herbicides, such as sulfonylureas and imidazolinones [104,128]. Studies have revealed that specific amino acid substitutions in the *ALS* gene can confer herbicide resistance to plants [129]. By using a CBE to target mutations in the base sequences corresponding to proline at position 171 (P171) and glycine at position 628 (G628) of the rice *ALS* gene, researchers obtained a series of *ALS* inhibitor-like herbicide resistance mutants (Figure 2b). Among them, P171S, P171A, P171Y, and P171F showed different levels of resistance after they were sprayed with five different types of *ALS* inhibitor-like herbicides [130,131] (Table S1). In addition, the triple-amino acid mutant P171F/G628E/G629S, in which there are mutations at the P171, G628, and G629 loci, showed high levels of resistance to all five *ALS* inhibitor-based herbicides [69]. Similarly, by altering specific base sites while aiming to maintain *ALS* activity, researchers are developing herbicide-tolerant lines of many plant species, including wheat [104], rice [69], maize [132], oilseed rape [62], tomato [133], watermelon [68], pear [134], and *Arabidopsis* [135] (Table S1). *ACCase* is a key enzyme in lipid biosynthesis. It represents the site of action for several commercially important herbicides, such as those of the aromatic phenoxy propionate (APP) class and the cyclohexanone (CHD) class [136]. Introducing a C2186R substitution in the rice *ACCase* gene via ABEs resulted in the production of haloxyfop-R-methyl-tolerant rice strains [137] (Table S1).

Other amino acid substitutions in ACCase confer resistance to rice haloxyfop, such as P1927F and W2125C, also identified through CRISPR-based screens [138,139]. In addition, editing *PPO* [140], *EPSPS* [141,142], *TubA2* [127], and *SF3B1* [143] has been reported to afford resistance to butafenacil, glyphosate, trifluralin, and herboxidiene (GEX1A) [144,145]. The advantage of base editing is that with targeted and precise substitution, more herbicide-resistant loci can be identified for agricultural production by constructing sgRNA libraries for gene screening (Figure 2a).

## 12. Multifunctional Single-Base Editors

Many important agricultural traits are associated with multiple heterogeneous base transformations [146,147]. To introduce both C-to-T and A-to-G substitutions in plant and mammalian cells, some teams have developed several new two-base editors by combining CBEs and ABEs. The two-base editors can efficiently generate mutations in two different bases simultaneously, enriching base editing tools and having important implications for species improvement and molecular evolution [148]. The team constructed a base editing system (GhBE3) for genetic transformation in cotton using cytosine deaminase (APOBEC1), Cas9 nickase (nCas9), and uracil glycosylase inhibitor (UGI). The system can efficiently and specifically introduce single-nucleotide mutations in cotton cells with high C–G to T–A single-base editing efficiency, with a C–T editing efficiency of 57.78%; this BE system will become a new and important tool for functional genomic research in cotton [61] (Table S1). In addition, the researchers constructed a novel saturation-targeted endogenous gene mutation BE, STEME; notably, the STEME double base editor can induce simultaneous C T and A G mutations at the target site with only one sgRNA guide, significantly increasing the saturation of base mutations in the target gene and the diversity of mutation types produced. By using STEME-1 and STEME-NG for targeted mutation of the rice *OsACC* gene, the researchers obtained an herbicide-resistant mutant [138] (Table S1). These two sets of STEMEs can accelerate trait development and function in any plant that can undergo CRISPR-based mutations. Based on the originally developed plant CT-CBE tool eCDAL, researchers constructed the double single-base editing vector pDuBE1 by fusing TadA-8e at the N-terminal end. pDuBE1 enabled double single-base editing at multiple loci in the rice genome. The application of pDuBE1 simultaneously induced point mutations in two herbicide-resistance-related genes, resulting in *OsALS*-P171F/*OsACC1*-I1781 V double mutant herbicide-resistant rice plants [148] (Table S1).

In addition, CBE can regulate gene expression by inserting premature stop codons at predetermined triplet codons via BEs by converting six codons—CAG, CGA, CAA, TCA, TAC, and TGG—into stop codons [149,150] (Figure 2d). Similarly, BE can also regulate gene expression by editing regulatory factors (Figure 2c). ACG or GTG may be substituted with ABEs for the initiation codon (ATG) [137,144,151]. This method of regulation of gene expression has been applied to a variety of plant species, including tomato, rice, wheat, and oilseed rape [144,152] (Table S1). Splicing eukaryotic mRNAs requires splice donor (GT) and splice acceptor (AG) sites, which can be disrupted by BEs. Therefore, by inducing misplacement, BEs can cause exon skipping [39], selective splicing, or intron retention. This method resulted in the generation of novel mutants in both *Arabidopsis* and rice [153]. We can expect that BE-mediated regulation of mRNA splicing and the introduction of the stop codon is also an approach to crop improvement [154].

## 13. Conclusions, Challenges, and Prospects

BEs can provide an effective and prospective genome editing approach for generating point mutations to control essential plant characteristics. With the creation of efficient multiple-base editing systems, efficient, precise, and targeted mutagenesis through genome editing sets the stage for the next generation of breeding strategies that will transform the future of agriculture [155]. In addition to the already discussed areas where base editing has been applied, base editing can also be used to edit organelle genomes [156,157,158,159,160]. The researchers split the DddAtox structural domain in the DddA protein into two parts, DddAtox-N and DddAtox-C, addressing its toxicity to mammalian cells, and added the mitochondrial guide peptide (MTS) gene. Following the successful development of an mtDNA editing system called DdCBE, the first molecular tool to enable precise editing of mtDNA, in 2022, researchers developed a new gene editing platform called transcription activator-like effector-linked dehydrogenase (TALED) [161,162,163]. TALED can perform A→G base conversion in mitochondria. The sterile lines needed to produce a good hybrid are cytoplasmic male sterility (CMS), which manifests as pollen abortive and is crossed with restorative lines to produce hybrid offspring [164]. Knockout of the orf79 gene in the mitochondria of the rice variety BTA and the orf125 gene in the mitochondria of the kale variety SW18 can restore fertility in sterile male lines using mitochondrial technology (mitochondria) [165]. The base editor is simple and precise and can be used to perform prescreens of laboratory experiments [161,162,163,165]. In addition, traditional breeding introduces resistance traits from wild species into cultivated species by crossbreeding, a method that takes years and is accompanied by unwanted mutations in traits other than the target trait [166,167]. Based on the understanding of the genetic and molecular laws of crop domestication, de novo domestication of naturally resistant wild plants using base editing techniques may be a novel strategy to obtain resistant crops [168,169].

However, several challenges remain to improve the efficiency of base editing and its applications in plant breeding. It should be pointed out that the past successes in plant breeding have been primarily built upon efficient exploitation of natural allelic variation at large numbers of loci existing in the crop germplasm resources. It remains a huge challenge to link different natural alleles at individual loci with phenotypic differences of target traits. This information is lacking and can help improve the efficiency of gene editing by narrowing specific genic region(s) for editing. Also, virtually all target traits, including yield potential, resistances or tolerances to abiotic and biotic stresses, and quality parameters, to be improved on by plant breeders involve large numbers of genes and complex gene networks. Thus, another challenge to improve the efficiency and effectiveness of base editing in plant breeding is how to accurately select target gene(s) to be edited, which depends on our knowledge of the genetic and molecular mechanisms underlying specific target traits and target genetic backgrounds to be improved. The greatest challenge, or question, is to what extent the gene editing technology applies to crops with large genomes, such as wheat and barley, because those crops have duplicated genomes or large numbers of duplicated genes, which make it even more difficult to select target genes and predict the consequences of products in addition to the transformation difficulty. Plant genome editing usually relies on Agrobacterium or gene gun-mediated introduction of sequence-specific nucleases such as CRISPR/Cas9 and selection marker expression frames into recipient plant cells, where the inserted exogenous genes can subsequently be isolated and removed from the chromosomes of the edited progeny plants by means of self or backcrossing. Agrobacterium transformation is more commonly used, but the process is time-consuming and laborious, especially for plants with complex ploidy, long breeding cycles, or asexual reproduction. Viral vector systems are ideal for the in vivo delivery and transient expression of exogenous genes and are an important complement to stable expression systems for transgenes [170]. However, there are still many important challenges to be overcome in the delivery system.

Technically, one constraint that limits base editing applications is the targeting scope of BEs, which rely on the PAM requirement of Cas proteins and the width of the catalytic reaction window [171,172,173]. To address this restriction, fusing deaminases with various Cas orthologous or engineered variants with altered or relaxed PAM specificities can be used to expand the editing scope, including SaCas9 [174], ScCas9 [175], SpCas9-NG [176], SpCas9-NRRH [177], SpCas9-NRTH [177] and so on. It’s worth noting that although using some variants has extended the range of base editors, it greatly reduces its editing efficiency and increases its dependence on target points. The SpRY variant is especially capable of targeting almost all PAMs, whereas the sgRNA editing frequencies showed extremely high results in low on-target editing frequencies. Therefore, further research is needed to improve the efficiency of base editors in maintaining the recognition of relaxed PAM.

In addition, whole-genome sequencing studies showed that BEs could generate genome-wide gRNA-dependent off-target mutations. Therefore, it is necessary to improve the editing specificity of ABEs and CBEs. Extending SgRNA guide sequences, using high-fidelity SpCas9 variants such as eSpCas9 [178], SpCas9-HF [179], and HypaCas9 [180], and delivering base editors as ribonucleoprotein complexes (RNPs) [181] were effective in reducing gRNA-dependent off-target effects. Besides, gRNA-independent off-target mutations were also found using CBEs but not ABEs in mice and rice. This may be due to the overexpression of deaminase, causing the whole genome, especially the gene enrichment region, to mutate randomly. Using an alternative deaminase to rAPOBEC1 or engineering the deaminase domain are effective strategies to reduce the off-target effects. For example, BE3 containing PpAPOBEC1, RrA3F, AmAPOBEC1, and SsAPOBEC3B were more specific than that containing rAPOBEC1 [182]. In rice cells, A3Bctd-VHM-BE3 and A3Bctd-KKR-BE3 exhibit markedly reduced gRNA-independent off-target editing. When editing target sites, BEs are often accompanied by unnecessary base substitutions [41,183]. One way to reduce the frequency of bystander mutations is to narrow the editing window. The width of the editing window is determined by the DNA base editor deaminase. Therefore, it takes lots of effort to modify cytidine deaminase by introducing amino acid mutations to narrow the editing window. YE1-BE3, YE2-BE3, EE-BE3 and YFE-BE4max based on

rAPOBEC1 with double or triple mutation reduced from a 5-nt editing window to 1–2 nts or 3 nts. However, to expand the application of BEs in plant genomes, base editors with expanded windows are also needed [25]. Therefore, an enriched toolbox of BEs developed from various Cas proteins linked with deaminases offers researchers numerous opportunities for functional genetic studies and crop breeding. While the combination of CBE and ABE can efficiently perform four base transitions (AT→GC, GC→AT), there is still a lack of more efficient editing tools (CGBEs are less efficient) for the other eight base transitions (AT→GC, GC→AT) and base insertion–deletions (indels)

Prime editing (PE) constitutes new precision gene editing tools developed by David Liu’s lab in 2019; the PE system consists of a Cas protein, reverse transcriptase, and pegRNA. pegRNA induces Cas9 nickase to cut the DNA strand and reverse transcriptase to synthesize a new DNA sequence from the unpaired pegRNA sequence, which is eventually integrated into the DNA to complete the gene editing. PEs can efficiently convert all 12 bases without relying on DSBs or donor DNA. They can also efficiently perform precise insertions (up to 44 bp) and deletions (up to 80 bp) of multiple bases [184]. PEs, therefore, constitute an all-purpose tool that enables major changes to the field of gene editing [185,186]. BEs allow single-base editing without creating DSBs, while the latest PEs not only induce all single-base mutations but also induce indels, and in this sense, PEs are more advanced than BEs [187]. However, PEs have more severe indel problems than do BEs. The detection of PE off-target sites still needs further confirmation. In addition, the safety of reverse transcriptase overexpressed in cells as a major building block remains an issue to be considered. Therefore, PE and BE functions complement each other to some extent. Ideally, we could first identify functional mutant loci by constructing a plant initiation editing library screen and then use BE for precise mutation targeting of the mutation. This integrated strategy would greatly facilitate direct gene evolution and germplasm innovation in crop improvement applications by targeting mutant loci through base editing. Gene editing technologies also present bottlenecks in crop improvement as current plant genetic transformation is inefficient; combining base editing techniques with traditional hybridization protocols to improve complex traits in crops is, therefore, a future trend [156,188]. It is hoped that, in the future, crops created by gene editing technology can be promoted for cultivation and create economic value in accordance with the policy on the regulation of gene editing technology and products.

## Data Availability

All of the data generated or analyzed during this study are included in this published article.

## Supplementary Materials

The following supporting information can be downloaded at: https://www.mdpi.com/article/10.3390/cimb45020059/s1, Table S1: Applications of base editing in plants.
