# Supplementary material for: Applications and Prospects of CRISPR/Cas9-Mediated Base Editing in Plant Breeding
Source: Curr Issues Mol Biol. 2023 Jan 19;45(2):918–35. doi: 10.3390/cimb45020059 (PMC9955079; doi:10.3390/cimb45020059)
Supplement: Supplementary file 1 [file cimb-45-00059-s001.zip › cimb-2150785-supplementary.pdf]

Table S1. Applications of base editing in plants.

Method	Plant species	Target gene	Gene function	Base editor construct	Trait improvement	Mutant base sites	Reference
ABE	Rice	*sgOs-siteG1*<br>*sgOs-site2*<br>*sgOs-site3*<br>*sgOs-site4*		ABE7.10-nSpCas9-NGv1	-	-	[19]
CBE	Rice	*OsSBEIIb*<br>*OsPDS*	*OsPDS:* encodes a phytoene desaturase *OsSBEIIb:* encodes a starch branching enzyme IIb in rice	PCXUN-be3	-	*OsPDS*:<br>G8→A8 G10→A10 G8→C8<br>*OsSBEIIb:*<br>G5,6→A5,6 G6→A6<br>G5→A5 G5→C5 G6→C6<br>G6→T6 G5→T5 G5,6→T5,6	[60]
CBE	Rice	*NRT1.1B*<br>*SLR1*	*SLR1* encodes a *DELLA* protein *NRT1.1B* encodes a nitrogen transporter	APOBEC1-XTEN-Cas9(D10A)-NLS	High nitrogen Use efficiency and Reduced plant height	*NRT1.1B:*<br>C7→T7(T327M)<br>*SLR1:*<br>C6→T6 (S97L)	[61]

GhBE3	Tetraploid cotton	*GhCLA*<br>*GhPEBP*	*GhCLA* functions in chloroplast development.<br>*GhPEBP* participates in the multiplex-branch	GhBE3	-	-	[61]
CBE	Rice<br>Wheat<br>Maize	RICE: *OsCDC48*<br>*OsNRT1.1B*<br>*OsSPL14*<br>Wheat：<br>*TaLOX2-S1*<br>*TaLOX2-S2*<br>*TaLOX2-S3*<br>Maize:<br>*ZmCENH3*	*OsCDC48* regulates senescence and cell death.<br>*NRT1.1B* encodes a nitrogen transporter.<br>*OsSPL14* controls grain yield and grain number	pnCas9-PBE	Reduced senescence and death.	*OsCDC48:*<br>C3→T3 C7→T7 C7→T7<br>C8→T8 C3,4→T3,4<br>C7,8→T7,8<br>C3,4,7→T3,4,7<br>*OsNRT1.1B:*<br>C4→T4 C7→T7<br>*OsSPL14:*<br>C3→T3 C6→T6<br>*TaLOX2:*C3,6,9→T3,6,9<br>C3→T3<br>*ZmCENH3:*C3→T3 C4→T4<br>C5→T5 C7→T7 C8→T8	[64]
ABE	Rice	*OsALS1*<br>*OsTubA2*<br>*OsGS1*<br>*OsACC*	*OsALS* encodes acetolactate synthase,<br><br>*OsTubA2*: a novel	TadA9	Herbicide resistance	*OsALS1*： A10→G10 (S627G)<br>*OsTubA2:*<br>T11→C11(M268T)<br>*OsGS1*： A11→G11 (H249Y)	[65]

			artificial rice germplasm resistant to dinitroaniline herbicides was produced<br><br>*GS1* gene is involved in regulating glutamine synthesis in old leaves<br><br>*ACC:* Herbicide tolerance			*OsACC:* T12→C12 (C2186K)	
CBE	tomato	*SlDDB1*<br>*SlDET1*<br>*SlCYC-B*	*SlDDB1*, *SlDET1* and *SlCYC-B*<br>: Responsible for the accumulation of carotenoids	PDicAID-nCas9-CDAUGI-LVA-NptII	Carotenoids accumulate more tomato strains	*SlDDB1:* G3→A3 A312T<br>G9→C9 (D310H)<br>G3→A3 and<br>G9→C9(D310H) and (A312T)<br>*SlDET1:*G2,3→T2,3 and<br>C9→T9 (P479F and A481V)<br>G2,3→T2,3 (P479F)<br>*SlCYC-B:* G3,4→A3,4 STOP codon<br>G6→A6 (M117I)	[66]

ABE	Strawberry	*FvebZIPs1.1*	the conserved uORF of the strawberry transcription factor gene *FvebZIPs1.1*	A3A-PBE	Utilize bases Edit fine-tune the sugar content of strawberries	-	[67]
CBE	Water melon	*ALS*	Acetyl lactate synthase (*ALS*) catalyzes the conversion of pyruvate to acetolactate during plant growth, leading to the biosynthesis of branched-chain amino acids.	CBE3	herbicide-resistant	C7C8→T7C8(P190S)	[68]
CBE	Water melon	*ALS*	Herbicide tolerance	pBSE901	Herbicide-resistant watermelon strains	C7C8→T7C8 (P190S) (Herbicide resistant) C7C8→T7T8 (P190L) （Unknown）	[68]

CBE	Oilseed rape	*BnALS1*	Herbicide tolerance	pnCas9-PBE	Benzisulfuron Resistant rapeseed	C6,7→T6,7 (P197S) (Herbicide resistant)<br>C6,7,8→T6,7,8 (P197F) （Unknown）	[68]
CBE	Rice	*ALS*	Herbicide tolerance	H-BE3-*OsALS*-P171<br>pH-BE3-*OsALS*-G628	Herbicide resistance	H-BE3-*OsALS*-P171:<br>C6,7→T6,7 (P171S)<br>C6→T6 and C7→G7 (P171A)<br>C6,7→T6,7 and C8→A8 (P171Y)<br>C6,7,8→T6,7,8 (P171F)<br>C6,7,8,9,10→T6,7,8,9,10 (P171F and R172C)<br>pH-BE3-*OsALS*-G628:<br>G5,6,7→A5,6,7: (G628E/G629S)<br>Combinations of edits generated by simultaneous editing of *OsALS*-P171 and *OsALS*-G628:<br>G5,6,7→A5,6,7 and C6,7,8→T6,7,8: (P171F/G628E/G629S)	[69]

ABE	Rice	*OsSPL14*<br>*OsSPL17*<br>*OsSPL16*<br>*OsSPL18*	They control grain yield<br>and grain number	VQR-Cas9 (D10A)/<br>VRER-Cas9 (D10A)	High yield	-	[76]
CBE	Rice	*OsALS*<br>*OsEPSPS*	*ALS*: Herbicide tolerance<br>*OsEPSPS;* encodes a key enzyme in the synthesis of aromatic amino acids	A3A/Y130F-CBE-V01<br>A3A/Y130F-nCas9-NG-CBE-V01	Herbicide resistance	*OsALS:*<br>G5→A5 (D349N)<br>G12→A12 (D550N)<br>*OsEPSPS:*<br>C7,8→T7,8 (P173L)	[80]

CBE	Rice	*OsSPL14*<br>*OsSPL16*	They control grain yield<br>and grain number	A3A/Y130F-CBE-V01	elevated expression<br>of *OsSPL14* and *OsSPL16* was detected in these edited lines	These edits<br>had destroyed the *OsMIR156* binding sites in the mRNAs of<br>*OsSPL14* and *OsSPL16*, The M0-1979-6-1 has a homozygous deletion at the *OsSPL14*-sgRNA01 site and biallelic<br>base editing at the *OsSPL16*-sgRNA01 site. These mutations also abolished the *OsMIR156* binding sites in the mRNAs of *OsSPL14*<br>and *OsSPL16*	[80]
CBE	Rice	*OsGS3*<br>*OsGW2*<br>*OsGN1a*	control grain yield	A3A/Y130FCBE-V01	Increased particle length (due to *OsGS3* knockout) and particle<br>width increase (due to knockout *OsGW2*)	*OsGS3:* W→STOP<br>*OsGW2:* Q→STOP<br>*OsGN1a:* Q→STOP	[80]

ABE	Rice	*SLR1*	*SLR1* could block its GA-dependent degradation, making the plant dwarf	pRABEsp-OsU6	plant dwarf	*SLR1:*<br>T6→C6 (V92A)	[81]
ABE	Rice	*OsSPL14*<br>*OsSPL17*<br>*OsSPL16*<br>*OsSPL18*	They control grain yield<br>and grain number	pRABEsp-OsU6sa	High yield	-	[81]
ABE	Rice (Indica rice)	*Waxy*	*Waxy* genes (*Waxy*): control straight-chain precipitation in rice	pHPABE-7-esgRNA	amylose content decreased	transgenic Mutant 1 to 3 (MT1, MT2 and MT3)<br>MT1 A1246G(N247D)<br>MT2 A1634G(N306D)<br>MT3 A496G(Q128R)	[91]

CBE	Rice	*Wx*	*Waxy (Wx):* control straight-chain precipitation in rice	PBETS1<br>PBE-TS2<br>PBE-TS3	Low amylose rice	PBE-TS1:<br>*Wxm5:*<br>C2,3,5→T2,3,5<br>(P124F and R125W)<br>PBE-TS2:<br>*Wxm6:*<br>G6,7→A6,7(G159K)<br>*Wxm7:*<br>G6→C6(G159A)<br>*Wxm8:*<br>G1→A1 and G6→C6<br>(G159A and D161N)<br>*Wxm9:*<br>G4→T4 and G6→A6<br>(G159E and V160F)<br>PBE-TS3:<br>*Wxm10:*<br>C5,6→T5,6(T178I)<br>*Wxm11:*<br>C5→G5 and C6→T6(T178S)	[92]

CBE	Wheat	*TaALS*	*TaALS* encodes acetolactate synthase, which functions in the biosynthesis of the branched amino acid.	A3A-PBE	Herbicide resistance	C7,8,9→T7,8,9 (P197F)	[104]
CBE	Brassica napus	*BnALS1* *IAA7* *RGA*	*BnALS1:* strong resistance of sulfonylurea herbicides, *IAA7,* and *RGA:* plant dwarfing to varying degrees	A3A-PBE	Herbicide resistance Dwarfed strains	*BnALS1:* C7,8→T7,8 (P179F R180C) *IAA7:* C3,4,6,7→T4,6,7 (P92F P92L) *RGA:*C3,4,7→T3,4,7 (P94L S95L)	[110]
CBE	Rice	*Pi-d2*	*Pi-d2:* Rice fungus resistance gene	hAID*Δ-XTEN-Cas9n-NLS (rBE5)	Blast resistance	G4→A4(M441I)	[114]

CBE	Rice	*OsSWEET14*	*OsSWEET14:* Susceptibility genes for white leaf blight	eAFID-3	the predictable multi-nucleotide-targeted deletions generated by AFID-3 outside the TATA box in the effector-binding element conferred enhanced resistance to bacterial blight without affecting plant growth	AFID-3-induced predictable deletions in *OsSWEET14*	[120]
ABE	Rice	*OsTubA2*	Rice α-tubulin gene *OsTubA2*	rBE14	A new artificial rice germplasm resistant to trifluraline and no loss of adaptation was obtained	T7→C7(M268T)	[127]

CBE	Maize	*ZmALS1*<br>*ZmALS2*	Acetyl lactate synthase (*ALS*): catalyzes the biosynthesis of branched-chain amino acids during plant growth.	NLS-APOBEC1-XTEN -nCas9(D10A)-UGI-SV40NLS (CT-nCas9)	Herbicide resistance	C7→T7 (P165S)<br>C7→G7 (P165A)<br>C7C8→T7G8 (P165W)	[132]
CBE	Tomato	*ALS*	Acetyl lactate synthase (*ALS*): catalyzes the biosynthesis of branched-chain amino acids during plant growth.	pDeSpnCas9-NG-PmCDA1-UGI （Target-AID）	Herbicide-resistant	C14→T14 (P186S)<br>C14→A14 (P186A)<br>C14→G14 (P186T)	[133]

CBE	Apple<br>pear	*ALS* and *PDS*	*ALS:* Herbicide tolerance<br>*PDS:* Carotenoid biosynthesis	pDenCas9_<br>PmCDA1_UGI	Anti-clonalulfuron and albino lines are obtained in pears.	*ALS:*<br>apple:<br>C12,13→T12,13:(P185L)<br>C11→T11(R186W)<br>pear: C14→T14<br>andC13→G13(P192C)<br>C13,14→T13,14 (P192F)<br>*PDS:*<br>apple C17→T17 Q153STOP<br>pear C11→T11 Q353STOP	[134]
ABE	Arabidopsis	*FT*<br>*PDS3*	*FT* is an integrator factor and a key gene for regulating flowering pathways in plants<br>*PDS:* Octahyd rolycopene dehydrogenase gene, which has the effect of protecting chlorophyll from photobleaching	pcABE7.10	Transgenic Arabidopsis strains with late-flowering and albino phenotypes were obtained	*PDS:* A6→G6 Incorrect splicing of RNA transcripts<br>*FT:* T7→C7 (Y85H)	[135]

ABE	Rice	*ACC*	*ACC:* Herbicide resistance	Ubi-1-ecTadA-ecTadA* -nCas9-3xNLS	Herbicide resistance	T7→C7 (C2186R)	[137]
STEME	Rice	*OsACC*	Herbicide tolerance	APOBEC3A–ecTadA–ecTadA* nCas9 (D10A)	Herbicide resistance	P1927F<br>W2125C<br>S1866F<br>A1884P	[138]
CBE<br>ABE	Rice	*OsACC*	herbicide resistance	eBE3<br>eCDA<br>eABE	Herbicide tolerance	*OsACC-T1*<br>T7→C7 (C2186R)<br>T13→C13 (C2186R)<br>A16→G16 (I1879V)	[139]
CBE	Rice	*EPSPS*<br>*ALS*<br>*DL*	*EPSPS, ALS:* Herbicide tolerance<br>*DL:* drooping leaf genes	NGv1 (D10A)	Herbicide tolerance	-	[142]
CBE	Rice	*EPSPS*<br>*ALS*<br>*DL*	Herbicide tolerance	Target-AID-NGv1 (D10A)	Herbicide tolerance	-	[142]

CBE	Rice	*ALS*	Acetyl lactate synthase (*ALS*): catalyzes the biosynthesis of branched-chain amino acids during plant growth.	nCas9Os-PmCDA1At (Target-AID)	Herbicide-Resistant	C3→T3 (A96V)	[144]
CBE	Tomato	*DELLA*	*DELLA* gene family: Regulation of gibberellin signaling in plants	nCas9At-PmCDA1Hs nCas9At-PmCDA1At (Target-AID)	-	nCas9At→PmCDA1Hs: C1→T1(PtoL) C3→G3(LtoV)	[144]

CBE	Tomato	*ERT1*	*ERT1:* regulate plant hormone signaling	nCas9At-PmCDA1At-2A	-	C5→T5(AtoV)<br>G5→C5andG3→A3 (VtoL)<br>G3→A3andG5→A5 (VtoI)<br>G3→C3andG5→A5 (VtoI)	[144]
pDUBE1	Rice	*OsALS*<br>*OsACC*	Herbicide tolerance	pDUBE1	Herbicide tolerance	C8,9,10→T8,9,10 (P171F)<br>A16→G16 (I1899V)	[148]
CBE	Rice	*OsCERK1*<br>*OsSERK1*<br>*OsSERK2*<br>*ipa1*	Encodes receptor-like<br>kinase.	rBE3 （APOBEC1-XTEN -Cas9n-UGI-NLS）	Detect the efficiency of rBE3	rBE3:<br>*OsSERK1* G4→A4 (D428N)<br>*OsSERK2* G6→A6 (D433N)<br>rBE4:<br>*ipa1* C4→T4 (S918F)	[176]

-, not reported.

Reference

19. Negishi, K., et al., *An adenine base editor with expanded targeting scope using SpCas9-NGv1 in rice.* Plant Biotechnol J, 2019. **17**(8): p. 1476-1478.
60. Li, J., et al., *Generation of Targeted Point Mutations in Rice by a Modified CRISPR/Cas9 System.* Mol Plant, 2017. **10**(3): p. 526-529.
61. Lu, Y. and J.K. Zhu, *Precise Editing of a Target Base in the Rice Genome Using a Modified CRISPR/Cas9 System.* Mol Plant, 2017. **10**(3): p. 523-525.
64. Zong, Y., et al., *Precise base editing in rice, wheat and maize with a Cas9-cytidine deaminase fusion.* Nat Biotechnol, 2017. **35**(5): p. 438-440.
65. Yan, D., et al., *High-efficiency and multiplex adenine base editing in plants using new TadA variants.* Mol Plant, 2021. **14**(5): p. 722-731.
66. Hunziker, J., et al., *Multiple gene substitution by Target-AID base-editing technology in tomato.* Sci Rep, 2020. **10**(1): p. 20471.
67. Xing, S., et al., *Fine-tuning sugar content in strawberry.* Genome Biol, 2020. **21**(1): p. 230.
68. Tian, S., et al., *Engineering herbicide-resistant watermelon variety through CRISPR/Cas9-mediated base-editing.* Plant Cell Rep, 2018. **37**(9): p. 1353-1356.
69. Zhang, R., et al., *Generating broad-spectrum tolerance to ALS-inhibiting herbicides in rice by base editing.* Sci China Life Sci, 2021. **64**(10): p. 1624-1633.
76. Hua, K., et al., *Genome Engineering in Rice Using Cas9 Variants that Recognize NG PAM Sequences.* Mol Plant, 2019. **12**(7): p. 1003-1014.
80. Ren, Q., et al., *Improved plant cytosine base editors with high editing activity, purity, and specificity.* Plant Biotechnol J, 2021. **19**(10): p. 2052-2068.
81. Hua, K., et al., *Precise A.T to G.C Base Editing in the Rice Genome.* Mol Plant, 2018. **11**(4): p. 627-630.
91. Huang, L., et al., *Creating novel Wx alleles with fine-tuned amylose levels and improved grain quality in rice by promoter editing using CRISPR/Cas9 system.* Plant Biotechnol J, 2020. **18**(11): p. 2164-2166.
92. Xu, Y., et al., *Fine-tuning the amylose content of rice by precise base editing of the Wx gene.* Plant Biotechnol J, 2021. **19**(1): p. 11-13.
104. Zong, Y., et al., *Efficient C-to-T base editing in plants using a fusion of nCas9 and human APOBEC3A.* Nat Biotechnol, 2018.
110. Cheng, H., et al., *Base editing with high efficiency in allotetraploid oilseed rape by A3A-PBE system.* Plant Biotechnol J, 2021. **19**(1): p. 87-97.
114. Ren, B., et al., *Improved Base Editor for Efficiently Inducing Genetic Variations in Rice with CRISPR/Cas9-Guided Hyperactive hAID Mutant.* Mol Plant, 2018. **11**(4): p. 623-626.
120. Wang, S., et al., *Precise, predictable multi-nucleotide deletions in rice and wheat using APOBEC-Cas9.* Nat Biotechnol, 2020. **38**(12): p. 1460-1465.
127. Liu, L., et al., *Developing a novel artificial rice germplasm for dinitroaniline herbicide resistance by base editing of OsTubA2.* Plant Biotechnol J, 2021. **19**(1): p. 5-7.
132. Li, Y.M., et al., *Precise base editing of non-allelic acetolactate synthase genes confers sulfonylurea herbicide resistance in maize.* Crop Journal, 2020. **8**(3): p. 449-456.
133. Veillet, F., et al., *Transgene-Free Genome Editing in Tomato and Potato Plants Using Agrobacterium-Mediated Delivery of a CRISPR/Cas9 Cytidine Base Editor.* Int J Mol Sci, 2019. **20**(2).

134. Malabarba, J., et al., *New Strategies to Overcome Present CRISPR/Cas9 Limitations in Apple and Pear: Efficient Dechimerization and Base Editing.* Int J Mol Sci, 2020. **22**(1).
135. Kang, B.C., et al., *Precision genome engineering through adenine base editing in plants.* Nat Plants, 2018. **4**(7): p. 427-431.
137. Li, C., et al., *Expanded base editing in rice and wheat using a Cas9-adenosine deaminase fusion.* Genome Biol, 2018. **19**(1): p. 59.
138. Li, C., et al., *Targeted, random mutagenesis of plant genes with dual cytosine and adenine base editors.* Nat Biotechnol, 2020. **38**(7): p. 875-882.
139. Liu, X., et al., *A CRISPR-Cas9-mediated domain-specific base-editing screen enables functional assessment of ACCase variants in rice.* Plant Biotechnol J, 2020. **18**(9): p. 1845-1847.
142. Endo, M., et al., *Genome editing in plants by engineered CRISPR-Cas9 recognizing NG PAM.* Nat Plants, 2019. **5**(1): p. 14-17.
144. Shimatani, Z., et al., *Targeted base editing in rice and tomato using a CRISPR-Cas9 cytidine deaminase fusion.* Nat Biotechnol, 2017. **35**(5): p. 441-443.
148. Xu, R., et al., *Development of an efficient plant dual cytosine and adenine editor.* J Integr Plant Biol, 2021. **63**(9): p. 1600-1605.
176. Ren, B., et al., *A CRISPR/Cas9 toolkit for efficient targeted base editing to induce genetic variations in rice.* Sci China Life Sci, 2017. **60**(5): p. 516-519.
